# A group penalization framework for detecting time-lagged microbiota-host associations

**DOI:** 10.3389/fgene.2025.1504443

**Published:** 2025-03-03

**Authors:** Emily Palmer, Austin Hammer, Thomas Sharpton, Yuan Jiang

**Affiliations:** ^1^ Department of Statistics, Oregon State University, Corvallis, OR, United States; ^2^ Department of Microbiology, Oregon State University, Corvallis, OR, United States

**Keywords:** grouped variable selection, longitudinal microbiome data, parasite worm burden, sparsity pattern, time lag, zebrafish

## Abstract

There is rising interest in using longitudinal microbiome data to understand how the past status of the microbiome impacts the current state of the host, referred to as “time-lagged” effects, as these effects may take time to occur. While existing works used previous states of the microbiome in their analysis, they did not use methods that identify both the time-lagged associations and their corresponding time lags. In this article, we present a framework to identify time-lagged associations between abundances of longitudinally sampled microbiota and a stationary response (final health outcome, disease status, etc.). We start with a definition of the time-lagged effect by imposing a particular structure on the association pattern of longitudinal microbial measurements. Using group penalization methods, we identify these time-lagged associations including their strengths, signs, and timespans. Through simulation studies, we demonstrate accurate identification of time lags and estimation of signal strengths by our approach. We further apply our approach to find specific gut microbial taxa and their time-lagged effects on increased parasite worm burden in zebrafish.

## 1 Introduction

The study of the gut microbiome is critical for understanding health and disease (Gomaa, 2020; Afzaal et al., 2022). The gut microbiome is not a static system; it has complex dynamics and the composition shifts constantly (Gerber, 2014). Changes to the gut microbiome can occur due to diet, medical interventions (i.e., antibiotic usage), and health status, among other drivers. Sampling the microbiome longitudinally (at several points in time) uncovers potential temporal variations in the microbiome, which can provide a full understanding of the ecosystem (Grieneisen et al., 2023). The dynamic aspect of the microbiome/host relationship is understudied and there is a need for analytical approaches that can handle this kind of complex data.

While many studies have investigated the link between the gut microbiome and health outcomes using a static snapshot of the gut microbiome and host health status, there is a growing recognition of the importance of incorporating longitudinal data. By examining the dynamic structure of the microbiome and its associations over time, researchers can discern patterns that may not be apparent in single-time point analyses or that may take time to develop. A prior state of the gut microbiome may be as or more informative of the current health of a patient than the current state of the gut microbiome. These connections are not necessarily immediate and may take time to occur. We call the association of a previous state with the current state a time-lagged association or a time-lagged effect. One famous instance of the time-lagged microbiota-host association is the long-term health and disease outcomes that are associated with the infant microbiome (Sarkar et al., 2021).

Identifying time-lagged associations/effects helps uncover the unique dynamics of the microbiome. Biological responses to changes in the microbiome do not necessarily appear immediately. Instead, these responses may manifest after some lag, creating a time-lagged association. A biological response associated with some disruption or change in the microbiome may not be observable until weeks later. By pinpointing the timing of these lagged associations, we gain insight into when interventions could be introduced for maximum effect. This approach allows for better-informed, time-specific strategies in microbiome research, leading to more effective interventions and a deeper understanding of host-microbiome interactions.

Multiple studies have been conducted to identify time-lagged microbiota-host association. Wilmanski et al. (2021) linked low gut microbiome uniqueness and high relative *Bacteroides* abundance to decreased 4-year survival in older healthy adults using Cox proportional hazard regression models. In a study to identify associations between the gut microbiome and nestling weight and survival in wild great tits, Davidson et al. (2021) identified specific microbial ASVs associated with surviving to fledgling using data from day 8 post-hatch, as well as specific ASVs associated with non-survival. Luna et al. (2020) developed a joint modeling framework to detect associations between longitudinal microbiome count data and time-to-event outcomes. They applied this method to analyze longitudinal samples of pregnant women and found that a 10% increase of the genus *Prevotella* was associated with a 1.5-fold increase in hazard of delivery. These studies show the wide interest and great potential in using longitudinal microbiome data to understand how the past status of the microbiome impacts the current state of the subject. However, none of these studies have used methods that identify both the time-lagged associations and their corresponding time lags, although they included previous states of the microbiome in their analysis. These studies showcase the need for more tailored methods.

In this work, we introduce a novel framework that identifies the lags and associations of specific taxa with a response, utilizing penalized group selection methods. The group selection methods identify taxa as well as associated time points to form their specific lagged associations. We apply this framework to real data of longitudinally sampled zebrafish gut microbiome and host parasite infection (Hammer et al., 2024). These data were originally collected to investigate the links between the microbiome, parasitic infection, and intestinal metabolites. Hammer et al. (2024) found the amount of microbiome disruption in parasite-infected zebrafish was correlated with parasite infection severity. Our analysis further found genera that were also identified as microbial mediators for the metabolome by Hammer et al. (2024) as well as additional genera worthy of further exploration to understand the gut microbiome-parasite burden link.

## 2 Methods

### 2.1 Time-lagged effects

In this section, we formally define a time-lagged effect and its corresponding time lag. For each of the n subjects, we collect repeated measures on p covariates, $X_{1},\ldots ,X_{p}$ , each of which is measured T times, denoted by $X_{j}={\left(X_{j,1},\ldots ,X_{j,T}\right)}^{\prime }$ , j=1,…,p . In a high-dimensional setting, we have p≫n . In addition, we measure a response Y , recorded at the final time point T for each subject, thus denoted by $Y_{T}$ . The relationship between the repeated measures of the covariates $X_{1},\ldots ,X_{p}$ and the response $Y_{T}$ is modeled through a linear model or a generalized linear model in which

$$g\left\{E\left(Y_{T}|X_{1},\ldots ,X_{p}\right)\right\}=\beta_{0}+X_{1}^{\prime }\beta_{1}+\cdots +X_{p}^{\prime }\beta_{p},$$ (1)

where $\beta_{j}={\left(\beta_{j,1},\ldots ,\beta_{j,T}\right)}^{\prime }$ are the coefficients for $X_{j}={\left(X_{j,1},\ldots ,X_{j,T}\right)}^{\prime }$ and g(⋅) is a link function depending on the distribution of the response. For example, g(⋅) can be the identity function when $Y_{T}$ follows a normal distribution and the logit function when $Y_{T}$ follows a Bernoulli distribution.

**FIGURE 1:**
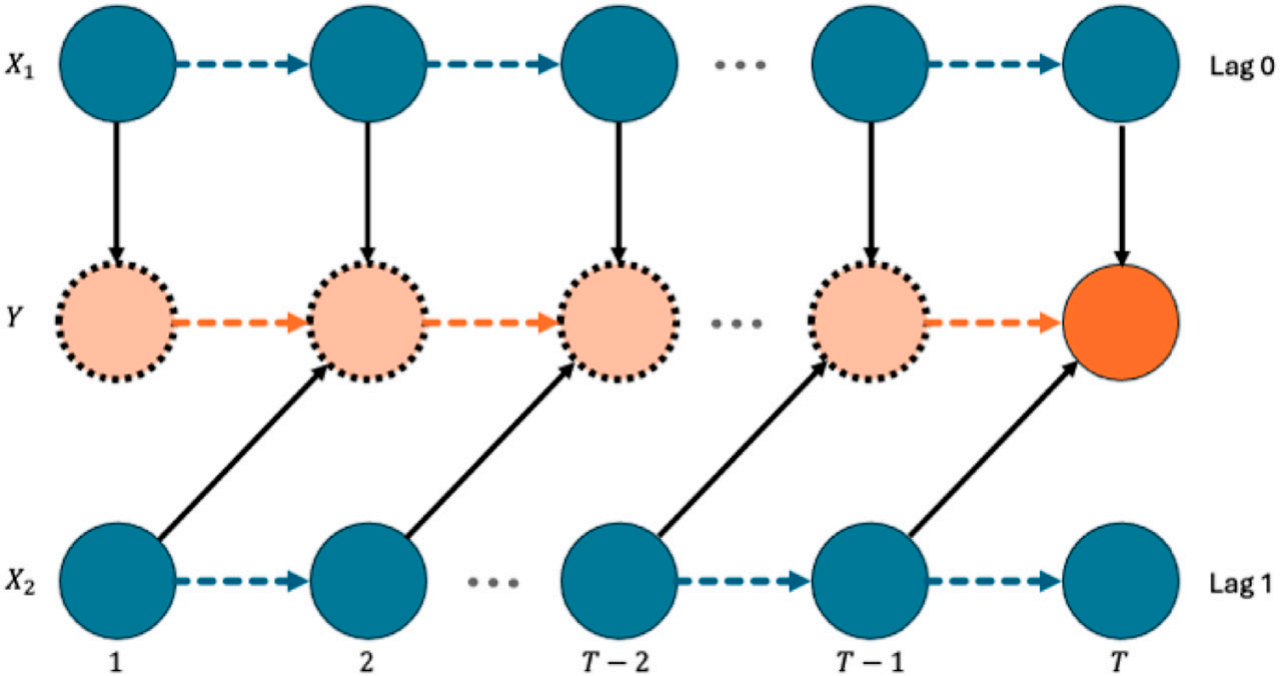
Diagram of time-lagged effects.

In this work, we focus on identifying and estimating time-lagged associations between the covariates and the response. We start with the definition of the time-lagged effect of a covariate on the response. To facilitate the definition, we illustrate the underlying relationship between two covariates and the response over time in Figure 1. In Figure 1, we lay out the repeated measures of two covariates $X_{1}$ and $X_{2}$ over time as well as the measurement of the response Y at the final time point T . We also include the hypothetical measurements of the response Y at previous time points t=1,…,T−1 to facilitate the interpretation of the time-lagged effect, although these measurements were not collected in practice (indicated by the dotted circles instead of solid circles in the diagram). The longitudinal dependence between the repeated measures of a covariate or between the repeated measures of the response is shown as dashed arrows, indicating the causal effect from a previous time point to the next. For simplicity of illustration, we did not include confounders for the repeated measures of covariates or the response in Figure 1, although adding them does not really change the interpretation of time-lagged effects.

In Figure 1, on the one hand, we say that $X_{1}$ has an instantaneous effect (with no time lag or lag 0) on Y , as shown by the solid directional arrows from $X_{1,t}$ to $Y_{t}$ at every time point t=1,…,T , as if $Y_{1},\ldots ,Y_{T-1}$ were measured. In this case, the instantaneous effect of $X_{1,T}$ on $Y_{T}$ , represented by $\beta_{1,T}$ in model 1, is nonzero. However, given that we do not observe the responses at the previous time points, $Y_{1},\ldots ,Y_{T-1}$ , we need to assess $\beta_{1,1},\ldots ,\beta_{1,T-1}$ in model 1, the effects of $X_{1,1},\ldots ,X_{1,T-1}$ on $Y_{T}$ . From the figure, it is seen that there are indirect associations between $X_{1,1},\ldots ,X_{1,T-1}$ and $Y_{T}$ through the longitudinal dependence between the repeated measures of either $X_{1}$ or Y . Even if we include all repeated measures of $X_{1}$ in model 1, the effects of $X_{1,1},\ldots ,X_{1,T-1}$ on $Y_{T}$ , represented by $\beta_{1,1},\ldots ,\beta_{1,T-1}$ , remain nonzero in model 1 due to the longitudinal dependence between the hypothetical repeated measures of the response. In summary, if $X_{1}$ has an instantaneous effect on Y , the coefficients in model 1) possess the following pattern:

$$\beta_{1,1}\neq 0,\ldots ,\beta_{1,T-1}\neq 0,\beta_{1,T}\neq 0.$$

On the other hand, $X_{2}$ has a 1-lagged effect on Y (time-lagged effect with lag 1), i.e., $X_{2,t}$ has a direct effect on $Y_{t+1}$ for any given time t=1,…,T−1 , indicated by the solid directional arrows from $X_{2,t}$ to $Y_{t+1}$ for t=1,…,T−1 . In this case, the 1-lagged effect of $X_{2,T-1}$ on $Y_{T}$ , represented by $\beta_{2,T-1}$ , is obviously nonzero. We still need to assess $\beta_{2,1},\ldots ,\beta_{2,T-2}$ and $\beta_{2,T}$ in model 1. Similar to $X_{1}$ , $\beta_{2,1},\ldots ,\beta_{2,T-2}$ are also nonzero in the model due to the indirect associations between $X_{2,1},\ldots ,X_{2,T-2}$ and $Y_{T}$ caused by the longitudinal dependence between the hypothetical repeated measures of the response. However, $\beta_{2,T}$ in 1 is zero, because the indirect association between $X_{2,T}$ and $Y_{T}$ is through $X_{2,T-1}$ , which has been included in the model. In summary, if $X_{2}$ has a 1-lagged effect on Y , the coefficients in model 1 possess the following pattern:

$$\beta_{2,1}\neq 0,\ldots ,\beta_{2,T-1}\neq 0,\beta_{2,T}=0.$$

Based on the above discussion, we formally define the time-lagged effect of a covariate $X_{j}$ on $Y_{T}$ with lag d through the following sparsity pattern of the corresponding coefficients $\beta_{j}={\left(\beta_{j,1},\ldots ,\beta_{j,T}\right)}^{\prime }$ :

$$\beta_{j,1}\neq 0,\ldots ,\beta_{j,T-d}\neq 0,\beta_{j,T-d+1}=0,\ldots ,\beta_{j,T}=0.$$ (2)

In other words, if $X_{j}$ has a d -lagged effect on $Y_{T}$ , the first T−d coefficients are nonzero and the last d coefficients are zero, as it takes d time intervals for $X_{j}$ to impact $Y_{T}$ .

The time-lagged effect of microbiome on host status is not uncommon in practice. For example, our real data analysis identifies several microbial taxa in the gut that have a variety of lagged associations with zebrafish parasite worm burden. In particular, abundances of genus *Chitinibacter* were found to have a 29-day-lagged association with parasite worm burden, whereas abundances of genus *Mycobacterium* were found to have an instantaneous association with parasite worm burden. More details of such findings can be found in the real data section.

### 2.2 Grouped variable selection methods

Due to the correspondence between the time-lagged effect and the sparsity pattern of the grouped coefficients in (2), identifying and estimating time-lagged effects can be regarded as a grouped variable selection problem. To identify these effects, we aim to pinpoint which time points have non-zero coefficient values, indicating a true association with the response. This process parallels traditional variable selection, where the objective is to determine which covariates contribute to the model. By considering the measurements of a covariate at different time points as a group, we treat the identification of time-lagged effects as a grouped variable selection problem, often solved using group penalization approaches, ensuring that only the relevant time points are retained in the model.

In the group penalization framework, we estimate the parameters $\beta ={\left(\beta_{1}^{\prime },\ldots ,\beta_{p}^{\prime }\right)}^{\prime }$ in model 1 by minimizing a objective function of the following form:

$$Q\left(\beta |X,Y\right)=L\left(\beta |X,Y\right)+P\left(\beta \right),$$

where L(β|X,Y) is the negative log-likelihood function depending on the underlying model 1 and P(β) is a penalty function, with observations $X={\left({\left(X_{1,1}^{\prime },\ldots ,X_{1,p}^{\prime }\right)}^{\prime },\ldots ,{\left(X_{n,1}^{\prime },\ldots ,X_{n,p}^{\prime }\right)}^{\prime }\right)}^{\prime }$ and $Y={\left(Y_{1,T},\ldots ,Y_{n,T}\right)}^{\prime }$ of sample size n .

In this subsection, we briefly review existing grouped variable selection methods as well as their corresponding penalty functions P(β) by classifying them into two categories—group-level/bi-level selection methods and overlapping group selection methods.

#### 2.2.1 Group-level/bi-level selection methods

Group-level selection methods select groups of variables and bi-level selection methods select both groups of variables and individual variables within a group, to represent their associations with a response. A well-known representative of group-level selection methods is group lasso (Yuan and Lin, 2006), while bi-level selection methods include group bridge (GrBridge) (Breheny and Huang, 2009), group exponential lasso (GEL) (Breheny, 2015), and composite MCP (cMCP) (Huang et al., 2012).

The first half of Table 1 shows the penalty functions P(β) for the above-mentioned methods when they are applied to the groups of coefficients $\beta_{1},\ldots ,\beta_{p}$ in model 1, where λ and γ are tuning parameters. We fix the tuning parameter γ at its default values, where γ=1/2 for GrBridge, γ=1/3 for GEL, and γ=3 for cMCP. In addition, the MCP function in Table 1 is defined as

$${\text{MCP}}_{\lambda ,\gamma }\left(\theta \right)=\left\{\lambda \theta -\theta^{2}/\left(2\gamma \right)\right\}\times I\left(\theta \leq \gamma \lambda \right)+\frac{1}{2}\gamma \lambda^{2}\times I\left(\theta >\gamma \lambda \right),$$

where I is the indicator function.

**TABLE 1:** Penalties for grouped variable selection methods.

Type	Follows pattern in (2)	Method	Penalty function
Group/Bi-level	✗	GrLasso	$P\left(\beta \right)=\sum_{j=1}^{p}\lambda \|\beta_{j}{\|}_{2}$
		GEL	$P\left(\beta \right)=\sum_{j=1}^{p}\frac{\lambda^{2}}{\gamma }\left(1-\exp\left\{-\frac{\gamma }{\lambda }\|\beta_{j}{\|}_{1}\right\}\right)$
		cMCP	$P\left(\beta \right)=\sum_{j=1}^{p}{\text{MCP}}_{\lambda ,\gamma }\left(\sum_{t=1}^{T}{\text{MCP}}_{\lambda ,\gamma }\left(\left|\beta_{j,t}\right|\right)\right)$
		GrBridge	$P\left(\beta \right)=\sum_{j=1}^{p}\lambda T^{\gamma }\|\beta_{j}{\|}_{1}^{\gamma }$
Overlapping	✓	O-GrLasso	$P\left(\beta \right)=\sum_{j=1}^{p}\sum_{t=1}^{T}\lambda \|\beta_{j}^{\left(t\right)}{\|}_{2}$
		O-GrMCP	$P\left(\beta \right)=\sum_{j=1}^{p}\sum_{t=1}^{T}{\text{MCP}}_{\lambda ,\gamma }\left(\|\beta_{j}^{\left(t\right)}{\|}_{2}\right)$
		O-GrSCAD	$P\left(\beta \right)=\sum_{j=1}^{p}\sum_{t=1}^{T}{\text{SCAD}}_{\lambda ,\gamma }\left(\|\beta_{j}^{\left(t\right)}{\|}_{2}\right)$

Applying group-level selection methods to the groups of coefficients $\beta_{1},\ldots ,\beta_{p}$ yields a subset of groups that are associated with the response, within each subgroup all individual variables included in the model. Applying bi-level selection methods to the same groups of coefficients yields a subset of groups and further a subset of variables within each group that are associated with the response. Notably, neither group-level nor bi-level selection methods may result in the desired sparsity pattern in (2) that defines the time-lagged effects.

**FIGURE 2:**
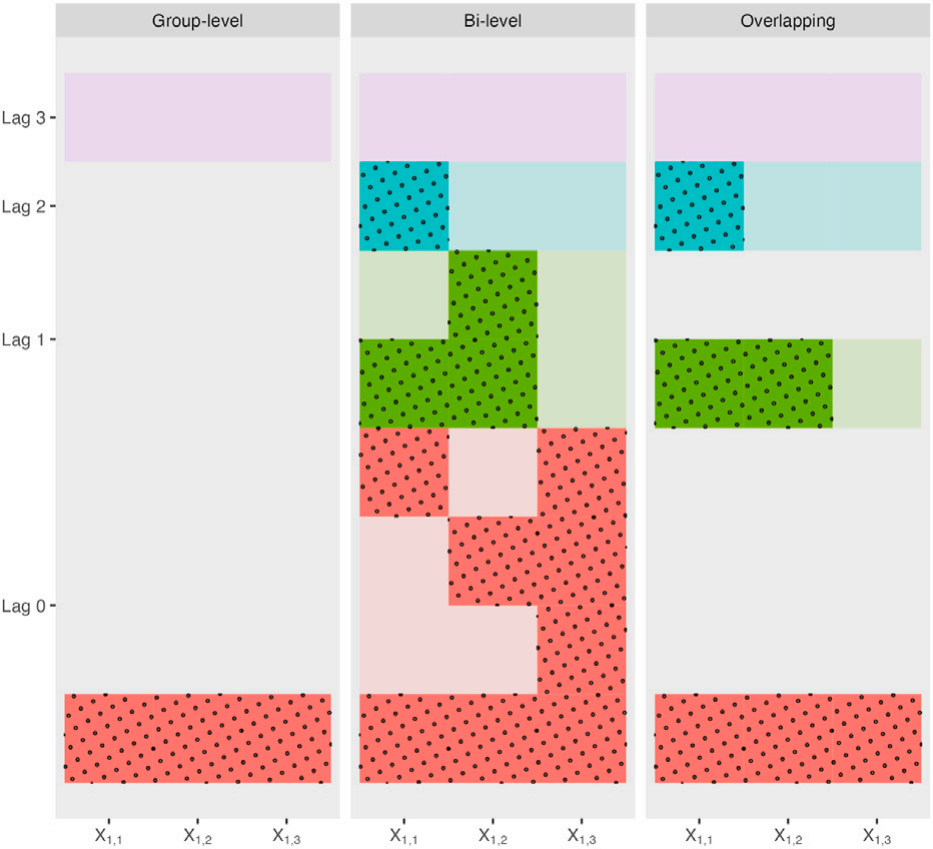
Possible sparsity patterns and their corresponding lags for each type of grouped variable selection methods applied to a toy example with one single variable $X_{1}$ measured at three timepoints. A filled-in (dotted) square indicates that variable/timepoint is present in the final model and a blank square indicates that variable/timepoint is absent in the final model.

Nonetheless, to report the performance of group-level/bi-level selection methods in lag identification, we still define the time lag for these methods using the largest index of nonzero estimated coefficients in a selected group. For example, for a variable $X_{1}$ measured 3 times, if a bi-level selection method provides zero-valued coefficients for $X_{1,1}$ or $X_{1,2}$ , or both, but a nonzero-valued coefficient for $X_{1,3}$ , we still define the estimated lag to be 0 for $X_{1}$ . Figure 2 shows the possible sparsity patterns as well as their corresponding lag definitions for group-level and bi-level selection methods applied to a toy example with a single variable $X_{1}$ measured at three timepoints.

#### 2.2.2 Overlapping group selection methods

Compared to the group-level/bi-level selection methods that are often applied to non-overlapping groups, overlapping group selection methods (Obozinski et al., 2011) impose group-level selection methods to overlapping groups. Interestingly, applying overlapping group selection methods appropriately yields the sparsity pattern that defines the time-lagged effect in (2), as illustrated below.

Instead of constructing one group of coefficients for a covariate $X_{j}$ in group-level/bi-level selection methods, i.e., $\beta_{j}={\left(\beta_{j,1},\ldots ,\beta_{j,T}\right)}^{\prime }$ , we construct T+1 groups for $X_{j}$ , $\beta_{j}^{\left(0\right)}={\left(\beta_{j,1},\ldots ,\beta_{j,T}\right)}^{\prime }$ , $\beta_{j}^{\left(1\right)}={\left(\beta_{j,1},\ldots ,\beta_{j,T-1}\right)}^{\prime }$ , … , $\beta_{j}^{\left(T\right)}=\emptyset$ , representing all possible sparsity patterns with lags 0,1,…,T , respectively. Once these groups are defined, overlapping group selection methods impose group-level penalization to these overlapping groups. Commonly used overlapping group selection methods include O-GrLasso, O-GrMCP, and O-GrSCAD, respectively imposing GrLasso, GrMCP, and GrSCAD to the overlapping groups (Jacob et al., 2009; Obozinski et al., 2011; Breheny and Huang, 2015).

The second half of Table 1 shows the penalty functions P(β) for the above-mentioned methods, where λ and γ are tuning parameters. It is noteworthy that these penalty functions involve the above-mentioned overlapping groups: $\beta_{j}^{\left(0\right)},\beta_{j}^{\left(1\right)},\ldots ,\beta_{j}^{\left(T\right)}$ for j=1,…,p . Similar to the group-level/bi-level methods, we fix γ at its default values, where γ=3 for O-GrMCP and γ=4 for O-GrSCAD. In addition, the SCAD function used in O-GrSCAD is defined as

$$\begin{array}{ll} {\text{SCAD}}_{\lambda ,\gamma }\left(\theta \right) & =\lambda \theta \times I\left(\theta \leq \lambda \right)+\left\{\gamma \lambda \theta -0.5\left(\theta^{2}+\lambda^{2}\right)\right\}/\left(\gamma -1\right)\times I\left(\lambda <\theta \leq \gamma \lambda \right)\\ & \;+\left\{\lambda^{2}\left(\gamma^{2}-1\right)\right\}/\left\{2\left(\gamma -1\right)\right\}\times I\left(\theta >\gamma \lambda \right). \end{array}$$

Due to the property of group-level selection methods, all its variables will be kept in the model if a group is selected. Therefore, for each selected group, its estimated coefficients satisfy the sparsity pattern in (2) due to the special construction of these groups. The final model for $X_{j}$ is the union of all selected coefficients from $\beta_{j}^{\left(0\right)},\beta_{j}^{\left(1\right)},\ldots ,\beta_{j}^{\left(T\right)}$ , which is just the largest selected group, also satisfying the sparsity pattern in (2). Therefore, the lag of the association of $X_{j}$ is well defined based on the largest index of nonzero coefficients in the final model. Figure 2 shows the possible sparsity patterns as well as their corresponding lag definitions for overlapping group selection methods applied to a toy example with a single variable $X_{1}$ measured at three timepoints.

## 3 Simulation

### 3.1 Simulation settings

To mimic the real data in our simulation, we make use of the microbiome data from a longitudinal study sampling the zebrafish microbiome (Hammer et al., 2024). The fecal microbiome of zebrafish was analyzed across three separate days (T=3) for 38 taxa (p=38) . After filtering for samples present in each of the 3 days, we are left with an initial sample size of 21 (n=21) . Measurements are then transformed using the centered log-ratio transformation (Aitchison, 1982) and serve as the covariates $X_{1},\ldots ,X_{p}$ . Details of this study and the data can be found in the real data section.

We simulate the response with two distributional settings, the normal distribution and the Poisson distribution. With the normal distribution, we simulate the response using a linear model: $Y=X_{1}^{\prime }\beta_{1}+\cdots +X_{p}^{\prime }\beta_{p}+\epsilon$ where ϵ∼N(0,1) . With the Poisson distribution, we set $\mu =\exp\left(X_{1}^{\prime }\beta_{1}+\cdots +X_{p}^{\prime }\beta_{p}\right)$ , and simulate the response using a Poisson model: Y∼Poisson(μ) .

For longitudinal data sampled at three time points, there are four possible lags, lag 0, 1, 2, and 3, where lag 3 corresponds to no association. Of the 38 taxa, we set a sparse signal of six true associations, two instances of each of lag 0, 1, and 2. We randomly assign the six true associations among the 38 taxa. Additionally, we test three magnitudes of the signal of the true associations, small, medium, and large. For the linear case these are $\beta_{j,t}\equiv c$ when $\beta_{j,t}\neq 0$ , where c∈{0.1,0.5,2} . For the Poisson case these are $\beta_{j,t}\equiv c$ when $\beta_{j,t}\neq 0$ , where c∈{0.01,0.05,0.1} . These values were chosen to simulate counts in a similar range to those present in the real data.

The original sample size of the zebrafish study (n=21) represents a small, but not unusual, sample size. We also want to test other sample sizes. To do this, we resample from the original 21 samples, and add noise from N(0,1) , resulting in a medium (n=50) and a large (n=100) sample size.

We compare seven grouped variable selection methods in two categories: group-level/bi-level selection methods and overlapping group selection methods. For the former, we applied group lasso (GrLasso), group exponential lasso (GEL), composite MCP (cMCP), and group bridge (GrBridge) to the simulated data. For the latter, we applied overlapping group lasso (O-GrLasso), overlapping group MCP (O-GrMCP), and overlapping group SCAD (O-GrSCAD).

### 3.2 Simulation results

We compare the performance of the seven grouped variable selection methods with various simulation settings: sample size (21,50,100) and signal magnitude (small, medium, large), for both linear model and Poisson model. The simulation results are summarized from 100 replicates. In the Poisson model, the GrBridge method did not converge in half of the simulation replicates in the medium-signal case when n=100 , and never converged in the large-signal case. Results are shown only for the cases in which the algorithm did converge.

#### 3.2.1 Group TPR and FPR

Since our goal is to identify the groups of variables associated with the response, we first examine the group true positive rate (TPR) and false positive rate (FPR). We define group rates based on whether at least one variable in the group is present in the model (regardless of whether it has the correct lag or not). Recall that we have six relevant groups with a signal (two repeats of each lag 0, 1, and 2); the remaining 32 groups are irrelevant as they have no signal. The group TPR and FPR are calculated based on the relevant and irrelevant groups, respectively.

**FIGURE 3:**
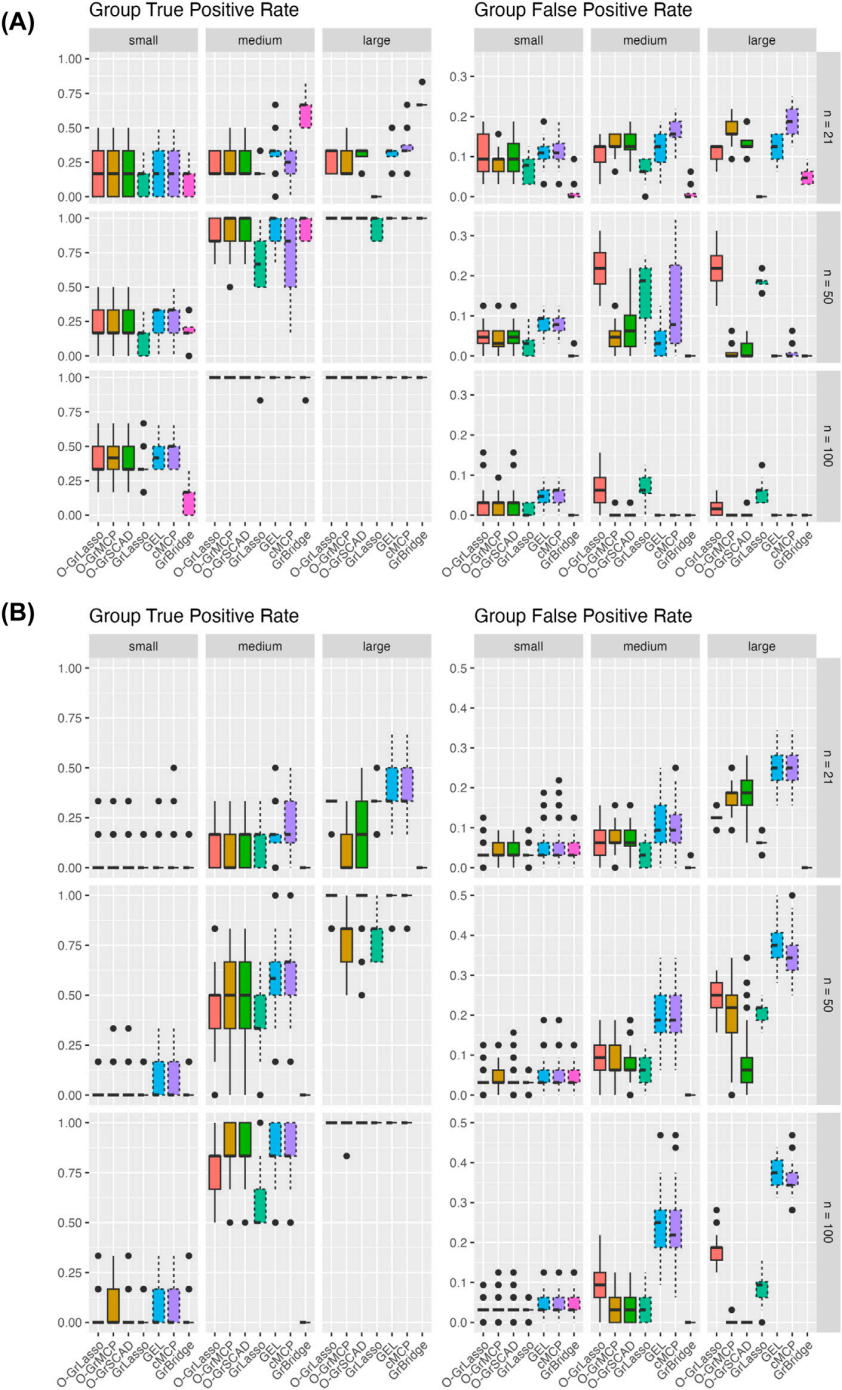
Group TPR and FPR for each method across three sample sizes (rows) and signal magnitudes (columns). Plot **(A)** shows results for the normal setting and plot **(B)** shows results for the Poisson setting. Solid line box plots represent the overlapping group selection methods; dotted line box plots represent the group-level/bi-level selection methods.

Figure 3 shows the group TPR (left) and FPR (right) across simulation settings. Comparing the normal and Poisson settings, group TPR and FPR have better performance in the normal setting than in the Poisson setting. GrBridge has one of the best performances in the normal setting, having the lowest group FPR in all cases, and comparable group TPR, but performs the worst in the Poisson setting, having very low group TPR even as the sample size increases.

Comparing the sample sizes, group TPR increase as the sample size increases in all cases. Group FPR also generally decreases with an increased sample size, but there are some cases of a higher group FPR when n=50 than when n=21 . This is likely because a larger possible model is allowed when n=50 so that more false positives slip through. The group FPR decreases again when n further increases to 100.

Comparing the signal magnitudes, group TPR remains lower with a smaller signal magnitude across all sample sizes and for both the normal and Poisson cases. We see group TPR increases as the signal size increases, however, we do not see the same trend for group FPR. The group FPRs in the normal setting are roughly the same as the signal magnitude changes. In the Poisson case we see somewhat a higher group FPR with a larger signal magnintude, but this can possibly be attributed to the increase of dispersion in the simulated responses.

Comparing the grouped variable selection methods, we generally see that the overlapping group and bi-level selection methods perform similarly to each other. The exception to this is GrLasso and O-GrLasso, which perform similarly, and worse than the other methods in the normal setting for a larger sample size and signal magnitude.

In the simulation setting that is the closest to the real data (the Poisson model with sample size of 21 and large signal magnitude), the group TPR is between 35% and 50% for a few methods such as O-GrLasso, GrLasso, GEL, and cMCP (see the right upper corner of the left panel in Figure 3B). In addition, in the same simulation setting, the group FPR is between 15% and 25% for the above methods (see the right upper corner of the right panel in Figure 3B). In other words, these few methods result in group TPR that are well above zero, and they control group FPR quite well. Such results suggest that these methods can identify potentially true signals from the real data analysis, although they may not be able to reveal all true signals for a data set of this size.

#### 3.2.2 Variable TPR and FPR

As we are also interested in seeing how well the methods perform in identifying the variables that should be present in the model, we additionally examine the variable TPR and FPR. Recall that in our simulation, we have 12 relevant variables and 102 irrelevant variables. The variable TPR and FPR are calculated based on the relevant and irrelevant variables, respectively.

**FIGURE 4:**
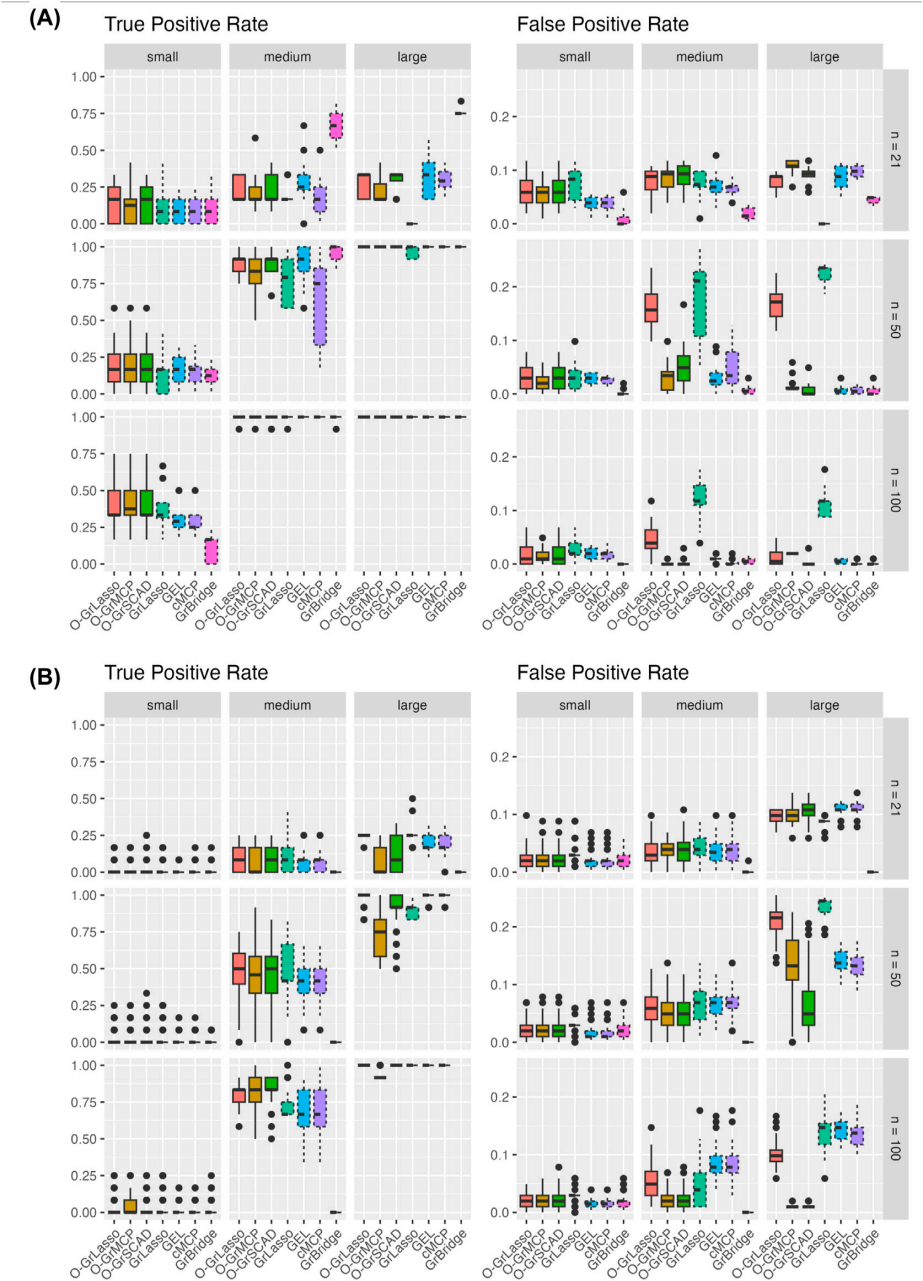
Variable TPR and FPR for each method across three sample sizes (rows) and signal sizes (column). Plot **(A)** shows results for the normal setting and plot **(B)** shows results for the Poisson setting. Solid line box plots represent the overlapping group selection methods, dotted line box plots represent the group-level/bi-level selection methods.

Figure 4 presents the variable TPR (left) and FPR (right) across our simulation settings. As the sample size increases, we see generally an increasing variable TPR and the rate becomes low in all cases except the small-signal case. GrBridge notably remains unlikely to pick up any of the true signals in the small-signal case, although it performs among the best in other signal cases for the normal setting.

Similarly, the variable FPR decreases when the sample size increases. In all settings, we see an average variable FPR below 0.25. GrLasso retains a higher FPR as the sample size increases, unsurprisingly, as it by definition always includes all variables of a chosen group, which is an overestimation if any non-instantaneous group is selected. We also see a slight increase in variable FPR when the sample size increases from 21 to 50, likely due to the increased allowable model size allowing more irrelevant variables to be included.

Comparing the grouped variable selection methods, we see a lower variable TPR from the bi-level methods in almost all cases. This is unsurprising as bi-level methods have the possibility of excluding relevant variables before the lag time as they do not necessarily maintain the correct sparsity pattern as in (2). Except GrLasso, the overlapping group selection methods and the bi-level selection methods yield comparable variable FPR and there is no obvious winner.

#### 3.2.3 Lag identification

**FIGURE 5:**
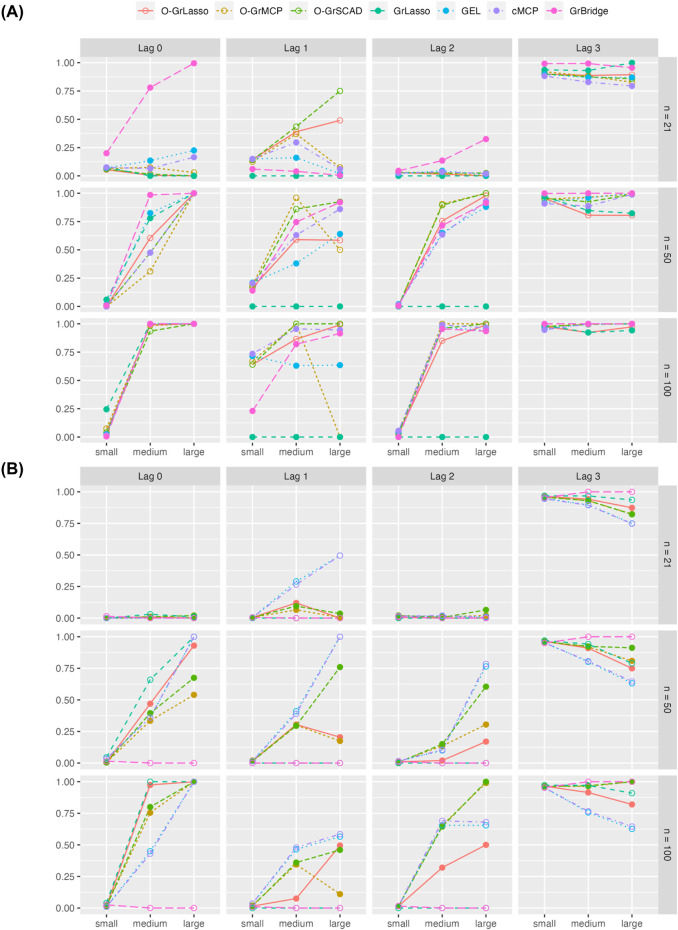
Proportion of correct lag-identification across simulation replicates for each of the four true lags. Plot **(A)** shows results for the normal setting and plot **(B)** shows results for the Poisson setting. Solid lines represent overlapping group selection methods, dotted lines represent the group-level/bi-level selection methods.

Figure 5 shows the proportion of the 100 replicates in which the lag is correctly estimated for each true lag (0–3). As expected, GrLasso performs the worst for intermediate lags of 1 or 2, as it can only ever identify all of the time points (lag 0) or none of them (lag 3) in a group.

Lags are generally more correctly identified as the sample size increases, although the proportion of correct lag-identification remains low in the setting with the smallest signal magnitude. Except in the setting with the smallest sample size and the smallest signal magnitude, all methods generally identify the correct lag over half of the time in the normal setting. The normal setting yields a higher proportion of correct lag-identification than the Poisson setting.

In general, lag identification also improves as the signal magnitude increases, although we see a few methods performing worse for true lag 1 as the signal magnitude increases. In the Poisson setting, an increased signal magnitude occasionally leads to a decreased proportion of correct lag-identification for true lags of 1 and 3.

Comparing the grouped variable selection methods, we do not see any clear winner between the overlapping group and bi-level selection methods. While an overlapping group selection method always identifies the sparsity pattern correctly whenever it identifies the lag correctly, it is not necessarily the case for a bi-level selection method. Therefore, we also report the proportion of incorrect identification of the sparsity pattern from the bi-level selection methods. Averaged across all sample-size and signal-magnitude settings, GEL has an incorrect lag pattern 22% of the time, cMCP has an incorrect lag pattern 29% of the time, and GrBridge has an incorrect lag pattern 12% of the time in the normal setting. In the Poisson setting, GEL and cMCP have an incorrect lag pattern 44% of the time, and GrBridge has an incorrect lag pattern 62% of the time. However, as we see from Figure 5, they can still generally identify the time lag correctly. These observations suggest a careful interpretation is needed for the time-lagged association from the bi-level selection methods.

Based on the simulation results, we have not identified a clear winner from the seven methods in all simulation settings. However, we could still make the following recommendations based on our limited observations. To ensure the sparsity pattern in (2), we recommend using the overlapping group selection methods, including O-GrLasso, O-GrMCP, and O-GrSCAD. In the simulation setting that is closest to the real data (Poisson model with sample size of 21 and large signal magnitude), O-GrLasso outperforms O-GrMCP and O-GrSCAD in terms of group selection and variable selection, suggesting its better performance in detecting the time-lagged effects. Nonetheless, we regard all these methods as a toolbox for identification of time-lagged effects and suggest the use of them in a complementary way.

## 4 Real data

### 4.1 Zebrafish data

We apply our group penalization framework to the real dataset from Hammer et al. (2024), which originally studied the role of the gut microbiome in mediating parasitic infection of *Pseudocapillaria tomentosa* in zebrafish. In our application, we make use of the data collected from longitudinally sampled zebrafish fecal samples (sampled on days 0, 3, and 32) to find time-lagged associations between the abundances of microbial taxa in the zebrafish gut and the parasite burden on zebrafish.

**FIGURE 6:**
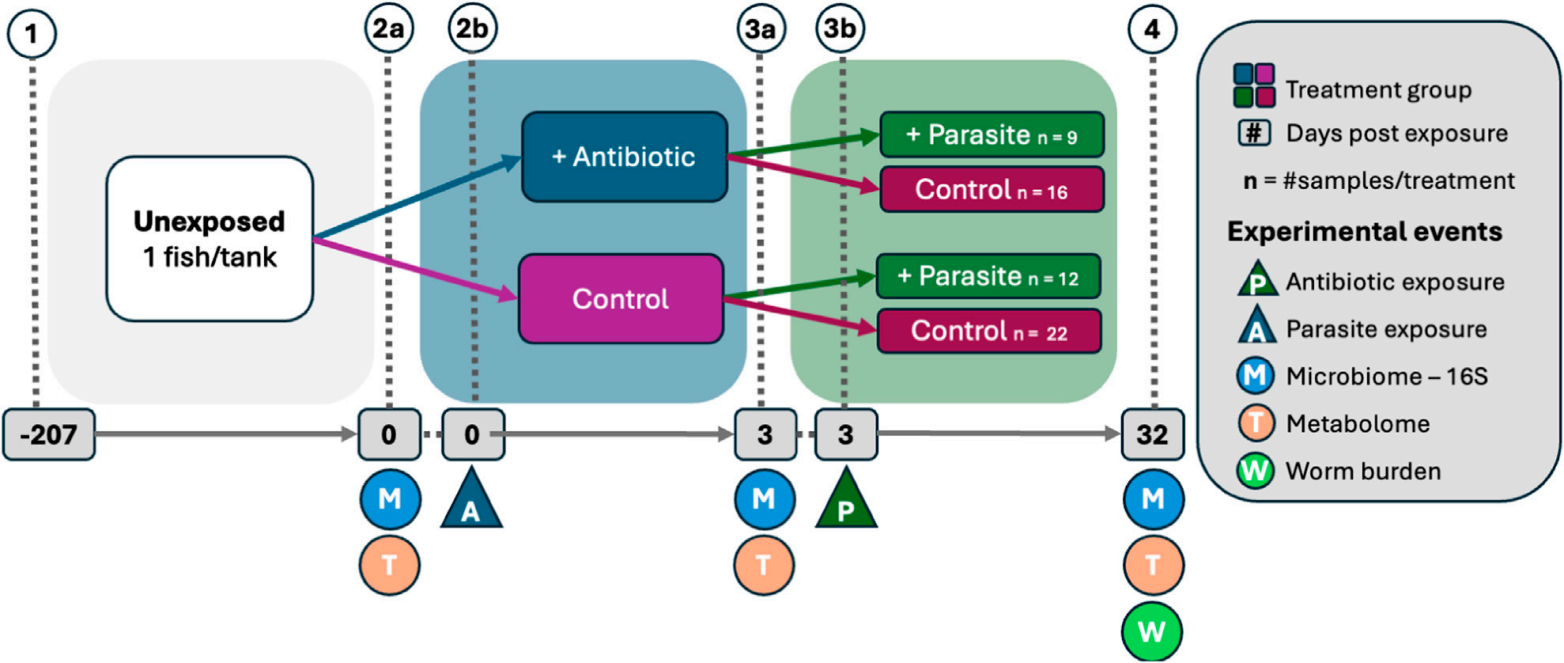
Schematic of real data experimental design by Hammer et al. (2024). (1) Adult fish were placed in individual tanks, (2b) half of the fish were exposed to antibiotics, (3b) fish were exposed to the zebrafish parasite *Pseudocapillaria tomentosa*. Only the parasite groups were used in the analysis with parasite worm burden as the response. Fecal microbiome and metabolome samples were collected (2a) prior to antibiotic exposure, (3a) prior to parasite exposure, and (4) 32 days post-antibiotic exposure after which fish worm burden was counted. Sample size represents fish alive throughout the study.

After day 0 and before day 3, half of the tanks of the zebrafish in the study were given an antibiotic and the other half were not. After day 3, in each group of zebrafish (antibiotic and control), roughly half of them were exposed to the parasite *P. tomentosa*. All zebrafish were sacrificed to assess intestinal histopathology on day 32, and the parasite burden on zebrafish was measured. In other words, microbiome data from the zebrafish gut were collected (a) prior to antibiotic exposure (day 0), (b) just prior to parasite exposure but after antibiotic exposure (day 3), and (c) 29 days post-parasite exposure (day 32). Figure 6 further explains the experimental design.

Since we use the final parasite burden as our response Y (day 32), our analysis only focuses on the half of fish that were exposed to the parasite. After filtering for die-off, 21 zebrafish remain in the parasite exposed group, 9 of which were given an antibiotic and 12 were not. In addition, we examine taxa at the genus level and apply an inclusion threshold to analyze genera present in at least 30% of samples, resulting in p=38 genera being included in our analysis.

### 4.2 Statistical analysis

In our analysis, the response Y is the final parasite burden on zebrafish, measured by the counts of the parasite *P. tomentosa* via intestinal histopathology. Therefore, we use a Poisson regression model for Y , with the link function in (1) chosen as the logarithm function. In addition, we apply a centered log-ratio transformation on the genus abundances collected on each day, and treat them as the repeated measures of the genera $X_{1},\ldots ,X_{p}$ where $X_{j}={\left(X_{j,0},X_{j,3},X_{j,32}\right)}^{\prime }$ for j=1,…,p .

We use these data to find time-lagged associations between final parasite burden of the zebrafish and genus abundances measured on day 0, 3, and 32. Since our samples are split into two groups, those exposed to antibiotics and those not exposed, we also take potential effects of antibiotic exposure into account in our modeling. To this end, we include antibiotic exposure as a main effect ( A , an indicator function for antibiotic exposure) as well as its interaction effects with the longitudinal genus abundances $\left(AX_{1},\ldots ,AX_{p}\right)$ , where $AX_{j}={\left(AX_{j,3},AX_{j,32}\right)}^{\prime }$ for j=1,…,p . It is noteworthy that there is no interaction between A and $X_{j,0}$ as the zebrafish are exposed to the antibiotic after day 0. In summary, the model in this data analysis can be written as:

$$\begin{array}{ll} Y|A,X_{1},\ldots ,X_{p} & ∼\text{Poisson}\left(\mu \right),\\ \text{where }\log\left(\mu \right) & =\alpha_{0}+A\alpha_{1}+X_{1}^{\prime }\beta_{1}+\cdots +X_{p}^{\prime }\beta_{p}+AX_{1}^{\prime }\gamma_{1}+\cdots +AX_{p}^{\prime }\gamma_{p}, \end{array}$$ (3)

where $\beta_{j}={\left(\beta_{j,0},\beta_{j,3},\beta_{j,32}\right)}^{\prime }$ and $\gamma_{j}={\left(\gamma_{j,3},\gamma_{j,32}\right)}^{\prime }$ for j=1,…,p .

We do not penalize the main effects $\alpha_{0}$ and $\alpha_{1}$ , and penalize the main effects $\beta_{1},\ldots ,\beta_{p}$ and the interaction effects $\gamma_{1},\ldots ,\gamma_{p}$ using either group-level/bi-level selection methods or overlapping group selection methods. For group-level/bi-level selection methods, a group of coefficients is formed for each taxon, ${\left(\beta_{j,0},\beta_{j,3},\beta_{j,32},\gamma_{j,3},\gamma_{j,32}\right)}^{\prime }$ for j=1,…,p . Group-level/bi-level selection methods then select from these groups and bi-level selection methods further select individual parameters in each group. For overlapping group selection methods, we construct 6 groups for each taxon j :

$$\emptyset ,\beta_{j,0},{\left(\beta_{j,0},\beta_{j,3}\right)}^{\prime },{\left(\beta_{j,0},\beta_{j,3},\beta_{j,32}\right)}^{\prime },{\left(\beta_{j,0},\beta_{j,3},\gamma_{j,3}\right)}^{\prime },{\left(\beta_{j,0},\beta_{j,3},\beta_{j,32},\gamma_{j,3},\gamma_{j,32}\right)}^{\prime },$$

Corresponding to different patterns of association with different lags. The first empty set corresponds to no association, the next three correspond to main effects with lags 32, 29, and 0 days, the last two correspond to main and interaction effects with lags 29, and 0 days. Note that the above construction of groups enforces the main effect to be included in the model before the corresponding interaction effects, whereas the bi-level selection methods have no such restriction.

### 4.3 Results

We tabulate in Table 2 the list of genera that are identified to be associated with parasite burden, together with their lags of such associations. For main effects, there are three possible lags—0, 29, and 32 days; for interaction effects, there are two possible lags—0 and 29 days, as there is no interaction between the antibiotic treatment and the microbial abundance on day 0. A lag of 0 days indicates an instantaneous effect, a lag of 29 or 32 days implies there is a lag for the microbial abundance to affect the parasite burden, either after antibiotic exposure or before antibiotic exposure. From the results in Table 2, we can draw the following conclusions.

**TABLE 2:** Lags of microbial main (M) and interaction (I) effects on worm burden (in days).

Genus	Overlapping group selection methods				Group/Bi-level selection methods							
	O-GrLasso/O-GrSCAD		O-GrMCP		GrLasso		GEL		cMCP		GrBridge	
	M	I	M	I	M	I	M	I	M	I	M	I
*Candidatus Accumulibacter*	0				0	0	0	0		0		
*Cetobacterium*			0				0					
*Chitinibacter*	29	29	29	29								
*Hyphomicrobium*	29	29	32						32			
*Legionella*									0			
*Mycobacterium*	0											
*Pelomonas*			0									
*Plesiomonas*									0			
*Pseudomonas*									32			
*Shinella*							29					
*Undibacterium*							32					

First, the results highlight the importance of identifying time-lagged associations. Five of the eleven identified genera have a time-lagged effect, which would not have been found if we were not using the proposed approaches.

Second, the overlapping group selection methods perform more similarly to each other, with O-GrLasso and O-GrSCAD producing the same results, while the bi-level selection methods do not have many similarities amongst themselves. GrBridge did not even identify any associated taxa. The most commonly identified genus was *Candidatus Accumulibacter* which was present in every method except O-GrMCP and GrBridge.

Third, we see a variety of identified lags. *Candidatus Accumulibacter* was identified with an instantaneous effect (lag of 0 days) by O-GrLasso/O-GrSCAD and GEL, *Chitinibacter* was found to have a lag of 29 days, including a 29-day lagged antibiotic interaction effect by all overlapping methods. *Hyphomicrobium* had a lag of 29 days by O-GrLasso and O-GrSCAD, and a lag of 32 days by O-GrMCP and cMCP.

Table 3 further presents the estimated coefficients for the identified genera in each model. Table 3 demonstrates a key difference between the overlapping group selection methods and the bi-level selection methods, namely, whether or not the sparsity pattern in (2) is met. For example, *Candidatus Accumulibacter* has a lag of 0 days for O-GrLasso and O-GrSCAD, as well as GEL, but GEL does not find an association on day 3, violating the sparsity pattern in (2). Additionally, *Candidatus Accumulibacter* is identified by cMCP to have an association with worm burden, but only the interaction effect on day 32, with no effects on earlier days and no main effects.

**TABLE 3:** Coefficient estimates of microbial main (M) and interaction (I) effects on worm burden.

Genus	Overlapping group selection methods														
	O-GrLasso/O-GrSCAD					O-GrMCP					GrLasso				
	M			I		M			I		M			I	
	$t_{0}$	$t_{3}$	$t_{32}$	$I_{3}$	$I_{32}$	$t_{0}$	$t_{3}$	$t_{32}$	$I_{3}$	$I_{32}$	$t_{0}$	$t_{3}$	$t_{32}$	$I_{3}$	$I_{32}$
*Candidatus Accumulibacter*	0.228	−0.001	0.08								−0.137	−0.048	0.136	0.013	0.304
*Cetobacterium*						−0.003	−0.361	0.563							
*Chitinibacter*	−0.290	0.120		0.507		−0.414	0.474		0.466						
*Hyphomicrobium*	0.663	−0.188		0.848		0.732									
*Legionella*															
*Mycobacterium*	0.045	−0.306	−0.35												
*Pelomonas*						0.099	−0.071	0.290							
*Plesiomonas*															
*Pseudomonas*															
*Shinella*															
*Undibacterium*															

Group/Bi-level selection methods														
GEL					cMCP					GrBridge				
M			I		M			I		M			I	
$t_{0}$	$t_{3}$	$t_{32}$	$I_{3}$	$I_{32}$	$t_{0}$	$t_{3}$	$t_{32}$	$I_{3}$	$I_{32}$	$t_{0}$	$t_{3}$	$t_{32}$	$I_{3}$	$I_{32}$
0.045		0.349		0.267					0.62					
	−0.114	0.379												
														
					0.449									
							0.424							
														
														
							0.146							
					−0.298									
	0.198													
0.329														

### 4.4 Connection with the original findings

The original paper (Hammer et al., 2024) including these zebrafish data conducted an analysis on the mediating role played by the gut microbiome on the relationship between gastrointestinal metabolites and parasitic infection outcome. This section highlights how our findings can further inform the longitudinal aspect of the relationship between the gut microbiome and parasite burden. Many of the genera the mediation study comments on as interesting are also found by our approach, and we expand upon these genera below.

Hammer et al. (2024) identified taxa in the *Pseudomonas* and *Mycobacterium* genera to be mediators in the relationship of the important Vitamin E metabolite γ -tocopherol and final worm burden. These genera are also present in our results. We found that abundance of *Pseudomonas* has a time-lagged and negative association with worm burden, where notably, the association only exists at the first time point (pre-antibiotic and parasite exposure). We also find that *Mycobacterium* has an instantaneous and negative association with worm burden and see that it gets stronger as the time is closer to the final time point.

Our findings also unite previous research which used these data by uncovering temporal changes in microbial association that help explain microbiota connections to parasite infection burden. For instance, results from Hammer et al. (2024) show that salicylaldehyde, which may be partially controlled by *Pelomonas*, has particularly strong effects on egg larvation and development. Our finding here that there exists an association between *Pelomonas* and infection burden on day 3 is both novel and important because this is the time fish hosts were exposed to parasite eggs and based on experimental evidence from stemming from this earlier work it is expected that salicylaldehyde-related inhibition of helminth maturation would be most pronounced at this time point in the study. Thus, this association from O-GrMCP points to time-dependent activity of *Pelomonas* that could help to explain and unite a connection between *Pelomonas* and salicylaldehyde to *in vivo* and *in vitro* results that implicate salicylaldehyde as an anthelmintic agent.

Additionally, results of applying these methods highlight a possible route by which gut microbes might regulate host intestinal structure to limit helminth infection, by regulating tight junction integrity. Results from both O-GrMCP and GEL point to day-3 associations in *Cetobacterium* are inversely related to helminth parasite infection. Prior work using the zebrafish model has shown that taxa within *Cetobacterium* synthesize vitamin B12 which mechanistically enhances gut barrier tight junction integrity to prevent microbial pathogen infection and improve gut microbiome structure stability (Qi et al., 2023). These findings are also relevant to nematode infection, where it has been shown using other infection models that early parasite exposure results in loss of epithelial barrier integrity as a result of changes in tight-junction related protein expression (Fernández-Blanco et al., 2015). Similar tight-junction regulating activity could underscore our results which point to early time points of *Cetobacterium* relative abundance negatively associating with parasite infection burden, potentially as a result of vitamin B12 biosynthesis.

Overall, we identify similar taxa associating with parasite worm burden as in Hammer et al. (2024), but our results provide important nuance to these findings by revealing time-dependent microbial associations with infection burden. For instance, our finding that *Pelomonas* is inversely associated with infection parasite burden on day 3 could help explain distinct results from Hammer et al. (2024) that revealed a connection between *Pelomonas* with salicylaldehyde, and salicylaldehyde to egg larvation. Furthermore, the connection between *Cetobacterium* and infection burden also uncovers a testable hypothesis regarding the relationship between microbes to metabolites and parasite infection that was not previously identified in the Hammer et al. (2024) analysis, and points to an additional route by which microbes could regulate helminth parasite infection. Together, clarifying the time during which a microbe might produce potent anthelmintic products or influence host response to infection can elucidate insights into the activity of microbes across the time range of parasite infection, possibly imparting new ways the gut microbiome may be harnessed to combat helminth parasite infection.

### 4.5 Metabolite validation analysis

To validate the microbial taxa that were identified to be associated with parasitic infection, we conduct a validation analysis using the additional measurements of metabolites in the zebrafish study (see Figure 6). We focus our analysis on the two metabolites that were found to be linked to parasite infection and whose effect on infection burden is mediated by members of the gut microbiome (Hammer et al., 2024), namely, salicylaldehyde and γ -tocopherol. We refer to Section 4.4 for detailed discussion on the role of these two metabolites in the microbiome-parasite ecosystem. Instead of worm burden used as a response, we now use each of the two metabolite measurements as the response and investigate the (potentially time-lagged) effects of microbial taxa on these metabolites. This additional analysis serves as the validation of the identified microbiome-parasite associations.

In the validation analysis, the response Y is the final metabolite measurement on day 32. Therefore, we use a Gaussian regression model for Y , with the link function in (1) chosen as the identity function. Metabolite measurements were log transformed. Since parasite burden is not included in this analysis, we include all groups of fish used in the initial study, i.e., using both the parasite and control groups. After filtering for die-off, we have 59 overall fish.

We use these data to find time-lagged associations between genus abundances measured on day 0, 3, 32 and final metabolite levels of the zebrafish measured on day 32. Since our samples are split into four groups, those exposed and not exposed to antibiotics, as well as those exposed and not exposed to parasites, we take potential effects of antibiotic and parasite exposure into account in our modeling. Compared to the model in (3), we include parasite exposure as an additional main effects ( P as an indicator function for parasite exposure). Thus, the model for the validation analysis can be written as:

$$\begin{array}{ll} Y|A,P,X_{1},\ldots ,X_{p} & ∼N\left(\mu ,\sigma^{2}\right),\\ \text{where }\mu & =\alpha_{0}+A\alpha_{1}+P\alpha_{2}+X_{1}^{\prime }\beta_{1}+\cdots +X_{p}^{\prime }\beta_{p}+AX_{1}^{\prime }\gamma_{1}+\cdots +AX_{p}^{\prime }\gamma_{p}, \end{array}$$ (4)

where $\beta_{j}={\left(\beta_{j,0},\beta_{j,3},\beta_{j,32}\right)}^{\prime }$ and $\gamma_{j}={\left(\gamma_{j,3},\gamma_{j,32}\right)}^{\prime }$ for j=1,…,p .

Similar to Section 4.2, we use bi-level/group-level selection methods and overlapping group selection methods to estimate the coefficients in (4). We do not penalize the main effects $\alpha_{0}$ , $\alpha_{1}$ , and $\alpha_{2}$ , and penalize the main effects $\beta_{1},\ldots ,\beta_{p}$ and the interaction effects $\gamma_{1},\ldots ,\gamma_{p}$ . The same groups of coefficients are formed as in Section 4.2 for group-level/bi-level selection methods and overlapping group selecion methods. The results of this validation analysis are presented in Tables 4–7. Tables 4, 5 show the time lags of microbial effects on salicylaldehyde and γ -tocopherol, respectively; Tables 6, 7 report the coefficient estimates of the above associations.

**TABLE 4:** Lags of microbial main (M) and interaction (I) effects on salicylaldehyde (in days).

Genus	Overlapping group selection methods						Group/Bi-level selection methods							
	O-GrLasso		O-GrSCAD		O-GrMCP		GrLasso		GEL		cMCP		GrBridge	
	M	I	M	I	M	I	M	I	M	I	M	I	M	I
*Acinetobacter*											0	0	0	0
*Aeromonas*													29	
*Bosea*											32	29		
*Bradyrhizobium*											29			
***Candidatus Accumulibacter***					**0**	**0**								
*Candidatus Odyssella*					0				0		0		0	
*Candidatus Protochlamydia*	32		32		32				32		0	29		
***Cetobacterium***											**29**			
*Chitinophaga*					32									
*Cloacibacterium*											29		0	29
*Dechloromonas*					32				32		29			
*Defluviimonas*												0		
*Dinghuibacter*													32	
Ensifer											29			
*Flavihumibacter*					0	0				29		29		
*Gemmata*					29				32		29		0	0
***Legionella***									**29**		**0**		**0**	**0**
***Mycobacterium***											**0**			
***Pelomonas***											**0**		**0**	
*Phenylobacterium*					32						32			
***Plesiomonas***													**32**	
*Rhodobacter*					0	0								
*Uliginosibacterium*													0	
*Vogesella*					0						29	0		

Genera in bold font are also identified in the parasite worm burden analysis.

**TABLE 5:** Lags of microbial main (M) and interaction (I) effects on γ -tocopherol (in days).

Genus	Overlapping group selection methods						Group/Bi-level selection methods							
	O-GrLasso		O-GrSCAD		O-GrMCP		GrLasso		GEL		cMCP		GrBridge	
	M	I	M	I	M	I	M	I	M	I	M	I	M	I
*Acinetobacter*					0	0			0	0	0		0	0
*Aeromonas*													0	
*Bosea*									32				32	
*Bradyrhizobium*											0			
***Candidatus Accumulibacter***											**0**			
*Candidatus Odyssella*											0			
*Candidatus Protochlamydia*											0			
***Cetobacterium***					**0**	**0**								
***Chitinibacter***										**0**				
*Cloacibacterium*					0	0			0				0	
*Dechloromonas*											32		32	29
*Defluviimonas*					0						0	0	0	29
*Dinghuibacter*									0	0	0	29		
*Ensifer*											0	0	29	
*Flavihumibacter*													29	0
*Flavobacterium*									0	0	29			
*Gemmata*													0	
***Legionella***					**0**				**0**	**0**	**0**		**0**	**0**
***Mycobacterium***											**29**			
***Pelomonas***											**29**		**0**	**29**
*Phenylobacterium*					0	0					0	0		
*Phreatobacter*												0		
***Plesiomonas***													**0**	**0**
***Pseudomonas***					**0**	**0**								
*Reyranella*											32			
*Rhizidiomyces*					32						0			
*Rhizobium*											32		32	0
*Rhodobacter*					0	0								
*Uliginosibacterium*									0	0	29			

Genera in bold font are also identified in the parasite worm burden analysis.

**TABLE 6:** Coefficient estimates of microbial main (M) and interaction (I) effects on salicylaldehyde.

Genus	Overlapping group selection methods														
	O-GrLasso and O-GrSCAD					O-GrMCP					GrLasso				
	M			I		M			I		M			I	
	$t_{0}$	$t_{3}$	$t_{32}$	$I_{3}$	$I_{32}$	$t_{0}$	$t_{3}$	$t_{32}$	$I_{3}$	$I_{32}$	$t_{0}$	$t_{3}$	$t_{32}$	$I_{3}$	$I_{32}$
*Acinetobacter*															
*Aeromonas*															
*Bosea*															
*Bradyrhizobium*															
***Candidatus Accumulibacter***						**−0.287**	**0.176**	**0.132**	**−0.216**	**−0.359**					
*Candidatus Odyssella*						−0.333	0.301	1.106							
*Candidatus Protochlamydia*	0.44					0.770									
***Cetobacterium***															
*Chitinophaga*						−0.260									
*Cloacibacterium*															
*Dechloromonas*						0.899									
*Defluviimonas*															
*Dinghuibacter*															
*Ensifer*															
*Flavihumibacter*						0.009	0.311	0.066	−0.758	0.014					
*Gemmata*						−0.531	−0.435								
**Legionella**															
***Mycobacterium***															
***Pelomonas***															
*Phenylobacterium*						−0.770									
***Plesiomonas***															
*Rhodobacter*						0.617	−0.258	0.284	0.699	−0.662					
*Uliginosibacterium*															
*Vogesella*						0.259	0.191	−0.092							

Group/Bi-level selection methods														
GEL					cMCP					GrBridge				
M			I		M			I		M			I	
$t_{0}$	$t_{3}$	$t_{32}$	$I_{3}$	$I_{32}$	$t_{0}$	$t_{3}$	$t_{32}$	$I_{3}$	$I_{32}$	$t_{0}$	$t_{3}$	$t_{32}$	$I_{3}$	$I_{32}$
							−0.168		0.398	−0.341	−0.041	−0.229	0.418	0.449
											−0.204			
					−0.117			0.151						
						−0.156								
														
		0.726					1.038					0.944		
0.443					0.520	0.411	−0.332	−0.645						
						**−0.276**								
														
					−0.087	−0.082				−0.385	−0.166	−0.422	0.569	
0.228					0.345	−0.146								
									0.254					
										−0.432				
						−0.141								
			−0.365					−0.580						
−0.321					−0.336	−0.142				−0.270	−0.355	−0.038	−0.312	−0.333
	**0.294**					**0.094**	**−0.248**			**0.099**	**0.031**	**−0.296**	**0.127**	**−0.714**
							**0.206**							
							**0.228**			**−0.358**	**0.235**	**0.207**		
					−0.346									
										**0.198**				
														
										−0.307		0.336		
					0.034	0.424			−0.032					

Genera in bold font are also identified in the parasite worm burden analysis.

**TABLE 7:** Coefficient estimates of microbial main (M) and interaction (I) effects on γ -tocopherol.

Genus	Overlapping group selection methods														
	O-GrLasso and O-GrSCAD					O-GrMCP					GrLasso				
	M			I		M			I		M			I	
	$t_{0}$	$t_{3}$	$t_{32}$	$I_{3}$	$I_{32}$	$t_{0}$	$t_{3}$	$t_{32}$	$I_{3}$	$I_{32}$	$t_{0}$	$t_{3}$	$t_{32}$	$I_{3}$	$I_{32}$
*Acinetobacter*						−0.100	0.262	−0.197	−0.093	−0.088					
*Aeromonas*															
*Bosea*															
*Bradyrhizobium*															
***Candidatus Accumulibacter***															
*Candidatus Odyssella*															
*Candidatus Protochlamydia*															
***Cetobacterium***						**0.273**	**0.076**	**−0.253**	**−0.161**	**0.176**					
*Chitinibacter*															
*Cloacibacterium*						0.360	0.216	−0.591	−0.683	0.247					
*Dechloromonas*															
*Defluviimonas*						0.073	−0.123	−0.240							
*Dinghuibacter*															
*Ensifer*															
*Flavihumibacter*															
*Flavobacterium*															
***Gemmata***															
***Legionella***						**0.013**	**0.003**	**−1.249**							
***Mycobacterium***															
*Pelomonas*															
***Phenylobacterium***						**−0.446**	**0.520**	**0.975**	**−1.663**	**−0.098**					
*Phreatobacter*															
*Plesiomonas*															
*Pseudomonas*						0.577	−0.024	0.372	−0.037	−0.934					
*Reyranella*															
*Rhizidiomyces*						0.324									
*Rhizobium*															
*Rhodobacter*						0.460	0.113	−0.516	−0.591	0.756					
*Uliginosibacterium*															

Group/Bi-level selection methods														
GEL					cMCP					GrBridge				
M			I		M			I		M			I	
$t_{0}$	$t_{3}$	$t_{32}$	$I_{3}$	$I_{32}$	$t_{0}$	$t_{3}$	$t_{32}$	$I_{3}$	$I_{32}$	$t_{0}$	$t_{3}$	$t_{32}$	$I_{3}$	$I_{32}$
−0.221	0.427	−0.492	−0.294	0.147		0.195	−0.323			−0.009	0.156	−0.270	0.014	0.294
											−0.320	0.314		
−0.351										−0.284				
					0.326		−0.180							
						**−0.067**	**0.015**							
						−0.191	0.125							
							0.393							
														
				−0.370										
		−0.285								−0.217	0.556	−0.307		
					−0.489					−0.514			0.136	
					0.239	−0.379	−0.367	0.187	0.171		−0.316	−0.122	0.176	
−0.658	0.066	−0.470	0.032	0.344	−0.556		−0.091	0.301						
					−0.303	−0.042	0.219	0.245	−0.090	−0.397	−0.199			
										−0.663	−0.120			−0.007
−0.246	1.197	0.088	−1.486	0.208		0.923								
										**−0.577**	**0.206**	**0.100**		
**0.217**	**−0.189**	**−1.189**	**0.740**	**0.655**		**0.040**	**−1.726**			**0.033**	**−0.127**	**−1.693**	**0.090**	**0.681**
					**−0.594**	**0.054**								
						−0.192				−0.265	−0.212	−0.129	−0.278	
					**−0.049**	**0.253**	**0.635**	**−1.435**	**0.431**					
									−0.069					
										−0.100		0.042		−0.479
														
					−0.479									
						−0.084	−0.162							
					0.250					−0.627			−0.306	−0.098
														
−0.624	0.295	0.054	0.008	0.293		0.860								

Genera in bold font are also identified in the parasite worm burden analysis.

Notably, of the eleven identified genera in the parasite burden analysis, eight were also identified and thus partially validated by the metabolite analysis. This result provides additional evidence for the critical role of the identified genera in the microbiome-metabolome-host relationship. Similar to Hammer et al. (2024), we also found an association between *Pelomonas* and salicylaldehyde, further supporting their relationship and joint roles in parasite infection burden as discussed in Section 4.4. Additionally, Hammer et al. (2024) identified *Pseudomonas* and *Mycobacterium* as mediators in the relationship between γ -tocopherol and final worm burden. We also identified *Pseudomonas* and *Mycobacterium* as associated with both final worm burden in the parasite burden analysis and γ -tocopherol in the metabolite validation analysis, validating the role of *Pseudomonas* and *Mycobacterium* as mediators in the relationship between γ -tocopherol and final worm burden.

## 5 Discussion

In this paper, we present a novel framework for identifying time-lagged associations between time-varying covariates and a static response, which enables the investigation of dynamics of host-microbiome interactions. Simulation studies demonstrate the efficacy of the framework in accurately identifying time-lagged associations.

Applying our framework to real zebrafish data further validated its utility. We identified eleven microbial taxa that exhibit associations with zebrafish parasite burden, four of which were instantaneous and seven others were lagged. Three identified taxa overlapped with those identified in the original study, two were instantaneous and one had a lag, reinforcing previous findings and highlighting new insights into time-lagged associations. For example, some associations changed their signs depending on the time lag, suggesting that the timing of intervention is as crucial as selecting the appropriate microbial target. The microbial taxa identified offer insights into potential mechanisms underlying the interplay between the gut microbiome and parasitic infections. This work contributes to a body of research that aims to clarify host-microbiome-parasite dynamics and informs future research toward developing targeted interventions for parasite control.

While this framework offers a practical approach to estimating time-lagged associations, there are a few limitations when using this framework. First, we argue that if there is an association present, it is measurable from the first time point sampled, and is present up until the lagged time point. In cases where this structure is not applicable, such as having only an instantaneous association, and no association from previous time points, the bi-level selection methods offer more flexibility in the temporal structure of which covariates are included. The definition of what it means to have a lag may need to be revisited or redefined depending on the context.

Second, another limitation relates to the length of available longitudinal data. Our method includes the entire timeframe of the data in search for lags, as our method assumes all prior covariates to the lag remain relevant. This assumption could be problematic if researchers are working with extended datasets spanning several years. The researchers would need to determine the reasonable timescale for a lag for their application. In some cases, a lagged association of 2 years would be reasonable, but in others only a 2-month lagged association would be reasonable.

Third, our framework uses different group penalization methods that can identify a set of interesting taxa. Future work can improve the prioritization of which of the model-identified taxa are interesting taxa to focus on. It is possible that different group penalization methods will identify either different sets of taxa or different lags, or both. Our framework encourages the researchers to use the set of identified taxa from the framework, but future work can help narrow down the focus.

Our application of this framework focused on advancing the understanding of microbial ecology and its influence on host health. However, this framework can be applicable to a much broader range of scientific fields, as it can be used whenever there is an interest in looking for time-lagged associations between longitudinal data and a static response.

## Data Availability

The nucleotide data underlying the findings of this study are available in the NCBI Sequence Read Archive (SRA) under BioProject ID PRJNA1132310, and annotated metabolomic data from positive and negative ion modes are available here (https://github.com/CodingUrsus/Zebrafish_Microbiome_and_Parasites/).
